# Exercise training modifies the bone and endocrine response to graded reductions in energy availability in skeletally mature female rodents

**DOI:** 10.3389/fendo.2023.1141906

**Published:** 2023-06-27

**Authors:** Susan A. Bloomfield, Sibyl N. Swift, Corinne E. Metzger, Kyunghwa Baek, Mary Jane De Souza, Scott Lenfest, Yasaman Shirazi-Fard, Harry A. Hogan

**Affiliations:** ^1^Bone Biology Laboratory, Department of Kinesiology & Sport Management, Texas A&M University, College Station, TX, United States; ^2^Bone Biology Laboratory, Department of Nutrition, Texas A&M University, College Station, TX, United States; ^3^Women’s Health and Exercise Laboratory, Department of Kinesiology, The Pennsylvania State University, University Park, PA, United States; ^4^Women’s Health and Exercise Laboratory, Department of Physiology, The Pennsylvania State University, University Park, PA, United States; ^5^Bone Mechanics Laboratory, Department of Mechanical Engineering, Texas A&M University, College Station, TX, United States

**Keywords:** caloric restriction, endurance exercise, IGF-1, leptin, estrogen, bone microarchitecture, histomorphometry, computed tomography

## Abstract

**Introduction:**

Reductions in energy availability leading to weight loss can induce loss of bone and impact important endocrine regulators of bone integrity. We sought to elucidate whether endurance exercise (EX) can mitigate bone loss observed in sedentary (SED) skeletally mature rodents subjected to graded energy deficits.

**Methods:**

Female virgin rats (n=84, 5-mo-old; 12/group) were randomized to baseline controls and either sedentary (SED) or exercise (EX) conditions, and within each exercise status to adlib-fed (ADLIB), or moderate (MOD) or severe (SEV) energy restriction diets for 12 weeks. Rats assigned to EX groups performed treadmill running to increase weekly energy expenditure by 10%. MOD-ER-SED, SEV-ER-SED, MOD-ER-EX and SEV-ER-EX were fed modified AIN93M diets with 20%, 40% 10%, and 30% less energy content, respectively, with 100% of all other nutrients provided.

**Results:**

Energy availability (EA) was effectively reduced by ~14% and ~30% in the MOD-ER and SEV-ER groups, respectively. MOD-ER for 12 weeks resulted in few negative impacts on bone and, except for serum leptin in MOD-ER-SED rats, no significant changes in endocrine factors. By contrast, SEV-ER in SED rats resulted in significantly lower total body and femoral neck bone mass, and reduced serum estradiol, IGF-1 and leptin. EX rats experiencing the same reduction in energy availability as SEV-ER-SED exhibited higher total body mass, lean mass, total BMC, and higher serum IGF-1 at the end of 12 weeks. Bone mechanical properties at 3 bone sites (mid-femur, distal femur, femoral neck) were minimally impacted by ER but positively affected by EX.

**Discussion:**

These findings indicate that combining increased EX energy expenditure with smaller reductions in energy intake to achieve a targeted reduction in EA provides some protection against loss of bone mass and lean mass in skeletally mature female rats, likely due to better preservation of circulating IGF-1, and that bone mechanical integrity is not significantly degraded with either moderate or severe reduced EA.

## 1 Introduction

Weight loss has been shown to negatively impact bone health. A comprehensive meta-analysis of clinical weight-loss trials in overweight/obese adults using moderate to severe energy restriction of 6-24 months duration found significant reductions in total hip areal BMD (aBMD, by 2-D DEXA) (0.010-0.015 g/cm^2^). This reduction represents a 1.0 - 1.5% decline in aBMD, roughly that lost yearly by post-menopausal women, and ~10-15% increase in fracture risk (1). A randomized clinical trial of weight loss in middle-aged men and women achieving a 20% caloric deficit by dieting only or increased exercise only demonstrated equivalent weight loss but loss of hip and spine aBMD only in the caloric restriction group (2). One 2-year trial utilizing moderate caloric restriction (-25%) in non-obese young adults found significant declines in a BMD at the lumbar spine and hip, along with increases in bone resorption markers and decreases in markers of bone formation (3). Therefore, studies demonstrate that weight loss/caloric restriction negatively impact bone regardless of starting weight.

The impact of reduced energy availability on bone integrity achieved concurrent with long-term regular weight-bearing exercise is less well described, but highly relevant to military and athletic populations. For example, unintentional weight loss is prevalent among military personnel. The rigors of basic training in recruits can require up to 5600 kcal per day (4), which level of energy expenditure is very difficult to replace with dietary intake. Weight loss has been identified as an important risk factor contributing to multiple health problems in military recruits, including the persistent issue of stress fractures (5), observed at much higher rates in female recruits (6). Whether or not regular weight-bearing exercise mitigates or exacerbates negative outcomes on bone in situations of high energy expenditure and low energy availability is not fully understood.

Crash dieting, weight cycling and disordered eating in women are known to negatively impact on reproductive hormone profiles, resulting in menstrual cycle disturbances and decreased bone mass. This cluster of outcomes has been particularly well described among some athletes who, intentionally or not, do not meet their energy needs over some months of time (7). [Recently, this concept has been updated to include male athletes as well, who can experience endocrine disruptions with more severe reductions in energy availability (8)]. Estrogen plays an important role in promoting bone mass accrual in adolescents and young adults and in maintaining bone mass in adults (9, 10). Its decline after menopause, along with co-existing aging changes like increased oxidative stress, is a prime contributor to the accelerated rate of bone loss post-menopause (11). Estrogen deficiency can interact with reduced energy availability when both occur, exacerbating the impact of either factor alone (12). Metabolic hormones also play an important role in regulating bone mass with fluctuations in energy availability, particularly IGF-1 and leptin (13).

Energy restriction protocols in appropriate animal models allow for more precise control over energy availability over longer periods of time, and enable quantification of changes in bone microarchitecture, geometry and mechanical properties and determination of cellular mechanisms. Numerous rodent studies have documented declines in bone mass (14–17) with dietary energy restriction. Also observed are declines in serum estradiol and 25-hydroxyvitamin D (18) and elevated corticosterone (19), as well as impaired mechanical properties of the femur (14, 17). The impact of energy restriction on skeletal health is particularly pronounced in young, rapidly growing mice, who also exhibit a doubling of bone marrow adipocyte density (20). However, all these studies were performed in sedentary animals restricted to cage activity; it is important to understand whether engaging in regular, vigorous exercise alters these documented impacts of energy restriction on bone integrity and endocrine mechanisms.

Previously, we found that exercise in energy-restricted rats mitigated bone loss but not endocrine changes observed in sedentary control rats (21). In a separate analysis, we reported that total body lean mass and total body bone mineral content were higher in exercised (vs. sedentary) rats after 12 weeks of reduced energy availability (22). Importantly, neither of our previous studies was able to comprehensively evaluate independent and interactive effects of exercise and reduced energy availability on site-specific bone density, microarchitecture, geometry, bone cell activity, and mechanical properties as compared to sedentary controls. Nor was a less severe level of reduced energy availability tested. Pivotal to these comparisons is achieving similar reductions in energy *availability* (kcal consumed minus caloric cost of exercise) in both sedentary and exercising animals over an extended period of time. This project was designed to test the hypothesis that skeletally mature rodents engaged in endurance exercise during graded reductions in energy availability would experience mitigated decrements in bone parameters important to fracture resistance (including directly measured mechanical properties) and in important endocrine regulators of bone integrity (IGF-1, leptin, and estradiol).

## 2 Methods

### 2.1 Animals and experimental design

Eighty-four virgin female Sprague-Dawley rats (5-mo-old) were purchased from Harlan-Teklad, group housed in a room with 12:12 hour light-dark cycles, and were switched off the vendor’s diet (Teklad 2018 non-purified diet) to the AIN-93M purified rodent diet (Research Diets D10012M, Research Diets, Inc., New Brunswick, NJ, USA) (23) for an 8-week acclimation period. Previous findings in our group revealed that adult female Sprague-Dawley rats experience significant declines in their proximal tibia cancellous vBMD over the first 4 weeks after switching from the Teklad rodent chow to the AIN-93M purified rat diet. Upon completion of this acclimation period, rats were moved to individual housing and assigned to one of seven groups (n=12/group) by random block assignment based on proximal tibia cancellous vBMD values (by pQCT) and body weight at Day 0. Those assigned to the baseline control (BC) group were terminated and tissues collected. The other six groups included adlib-fed control groups that were restricted to cage activity (ADLIB-SED) or subjected to exercise training (ADLIB-EX) designed to increase total weekly energy expenditure by 10%. Similar numbers of rats were assigned to two graded levels of energy restriction: -20% or −40% energy-restricted for sedentary rats (MOD-ER-SED and SEV-ER-SED, respectively), or -10% or −30% energy restricted for those assigned to exercise (MOD-ER-EX and SEV-ER-EX, respectively). Energy restriction was achieved by feeding animals custom-made variations of the AIN-93M diet with energy content of the diet reduced by 20% and 40% for sedentary rats and by 10% and 30% for exercised rats. The goal was to achieve equivalent graded reductions in energy availability (kcal consumed minus exercise kcal expended) in matched sedentary and exercised groups. All animal protocols were reviewed and approved by the Texas A&M Institutional Animal Care and Use Committee.

Body weights were recorded weekly to monitor animal health; vaginal smears were collected daily during the 2 weeks prior to Day 0 and from 10-12 weeks to determine estrus cycle status. Just before *in vivo* pQCT scans were performed on Day 0 and after 12 weeks, serum was collected from the saphenous leg vein in anesthetized animals for assays on insulin-like growth factor (IGF-1), leptin and estradiol at day 0 and after 12 weeks. These collections were timed according to each rat’s individual estrous cycle data to standardize collection of serum during the metestrus or early diestrus phase of each rat’s cycle. At the same time points but on a separate day, body composition and total body BMC was measured with dual energy x-ray absorptiometry (DEXA) scans under anesthesia. Calcein was injected subcutaneously (35 mg/kg) on days 9 and 2 prior to the end of the experiment to label mineralizing bone for dynamic histomorphometry. Animals were euthanized under anesthesia by cervical dislocation and then tissues were harvested. Right proximal tibiae were cleaned of all tissue and stored in 70% ethanol at 4°C until processed for histomorphometric analysis; the left tibiae and one whole femur were wrapped in saline-soaked gauze and stored at -35°C for *ex vivo* computed tomography and mechanical testing. Uteri were weighed after the removal of the ovaries and cervix, and any unusually high levels of fluid within the uterus were noted. Uterine weights were recorded as a bioassay for estrogen activity.

### 2.2 Dietary treatments

All rats were fed the AIN-93M diet (ADLIB groups) or custom-made variations based on AIN-93 M. Rats in the MOD-ER and SEV-ER sedentary groups were fed 0.81 and 0.61 gm, respectively, of a custom-formulated diet for every 1 gm of AIN-93M consumed by the average of both of ADLIB groups. Rats in the MOD-ER and SEV-ER exercise groups were fed 0.91 and 0.71 gm of a custom-formulated diet for every 1 gm of AIN-93M consumed by both ADLIB groups. Hence, the reductions in energy intake for exercised rats were smaller than those for sedentary animals to account for the increased caloric expenditure with treadmill training, with a goal of equalizing the energy availability across matched groups. The energy density of the restricted diets was altered by decreasing the total amount of corn starch; they also contained a higher density of all vitamins and minerals in order to achieve 100% of the animals’ daily requirements as established by the National Research Council, as previously described in detail (24) (see **Supplementary Table 1** for macronutrient information). Each animal’s food intake was measured daily and exercise energy expenditure verified (from exercise time and body weight) to document the daily energy availability for each animal. Energy available (EA) per week was quantified as total calories consumed over that week per animal minus any energy expended (kcal) via exercise during the same week per animal. EA was then normalized by lean body mass and represented as kcal/g lean body mass.

### 2.3 Treadmill exercise

Exercising groups were acclimatized to treadmill exercise for three weeks before the start of the experiment. During acclimation and before randomization, all rats were exercised 4 times per week on a 15% grade for incrementally increased periods of time and speed. For the 12 weeks of the dietary control period, rats assigned to exercise groups ran 4 days per week at 25 m/min on a 15% grade for 80-100 minutes. The duration of exercise was calculated weekly (based on body weight) in order to achieve a 10% increase in the animal’s energy expenditure. The intensity of this running protocol is approximately 70% of adult female rodents’ maximal oxygen consumption (25). Some rats needed mild stimulation to continue performing the prescribed exercise that was provided by a low-current electrical grid at the back of the treadmill belt or by short bursts of air from a modified air gun. Most rats adapted well to treadmill running and easily avoided the shock grid after the first training session.

### 2.4 Dual energy x-ray absorptiometry (DEXA)

Whole-body scans (GE-Lunar Prodigy Small Animal Program) were performed in the last two days before Day 0 and again two days prior to the end of the experiment on anaesthetized (ketamine/medetomidine, 2:1 ration, 0.5 mL/kg body weight) rats in order to assess body composition (lean and fat mass) and total body bone mineral content (BMC). Each rat was laid prone with its long axis aligned with the scan table’s center line. Regions of interest (ROI) were drawn to exclude the animals’ tails below the 3^rd^ caudal vertebrae. Coefficients of variance for *in vivo* determinations of lean mass, fat mass and total body BMC were ± 1.07, 2.99, and 1.24%, respectively, as determined from three repeat scans done on 4 separate rats with repositioning for each scan.

### 2.5 Peripheral quantitative computed tomography (pQCT)

#### 2.5.1 In vivo

At baseline and after 12 weeks, rats were anesthetized via vaporized isoflurane for *in vivo* scans of the proximal tibia metaphysis (mixed cortical and cancellous bone site) taken on a Stratec XCT Research-M device (Norland Corp., Fort Atkinson, WI). Metaphyseal volumetric bone mineral density (vBMD) was measured at the proximal tibia 4 slices distal from the growth plate. Three contiguous slices were averaged for one measure at the proximal tibia metaphysis. The region of interest therefore extended 2-3.5 mm distally from the growth plate. Scans were completed with 2.5 mm/sec scan speed, 100 μm voxel resolution, and 0.5 mm slice thickness. Measures obtained from the *in vivo* pQCT scans include cancellous vBMD and total vBMD (cancellous + cortical shell) and BMC.

#### 2.5.2 Ex vivo

After thawing, the femoral neck (another mixed bone site) was scanned using pQCT, using the same scan parameters as listed above. The proximal half of the femur was positioned so that the region of interest (1.5 mm wide) cut perpendicularly across the midpoint of the femoral neck, as ascertained with a scout view. Total bone mineral content (BMC) and volumetric bone mineral density (vBMD) were determined from the average of three contiguous scans.

### 2.6 Endocrine assays

Serum leptin was measured by a rat ELISA kit according to the manufacture’s protocol (Alpco Diagnostics, Salem, NH). The inter-assay coefficient of variation is less than 10% and the lowest detectable level was 10 pg/mL. Serum IGF-1 was measured using an EIA kit as suggested by the vendor (IDS Inc, Fountain Hills, AZ). The inter-assay coefficient of variation was 6% and the lowest detectable level was 2.8 ng/mL. Serum estradiol was measured using an estradiol double antibody RIA according to the vendor’s instructions (Siemens, Plainfield, IN). The inter-assay coefficient of variation was less than 6% and the lowest level detectable is 1.4 pg/ml.

### 2.7 Cancellous bone histomorphometry

Undemineralized proximal tibiae were serially dehydrated and embedded in methylmethacrylate (Aldrich M5, 590-9). Serial frontal sections were microtomed either 8 µm thick for analysis of calcein labels in unstained sections at 20X (total bone surface, single- and double-labeled surface, and inter-label distances) or 4 µm thick for von Kossa staining and measurement at 40X of static histomorphometric properties (bone volume, osteoid surface, osteoblast surface, osteoclast surface, and adipocyte density). The region of interest began ~1.0 mm distal to the growth plate and encompassed a 6.0 mm^2^ area within the endocortical edges. Mineral apposition rate (MAR; µm/day) was calculated by dividing the average inter-label width by the time between labels (7 days), and mineralizing surface (MS) for cancellous bone surfaces (BS) was calculated by using the formula MS/BS = [(single-labeled surface/2) + double-labeled surface]/surface perimeter x 100. Bone formation rate (BFR) was calculated with the formula: BFR = MAR X MS/BS. All histomorphometric analyses were performed using OsteoMeasure image analysis software (Version 2.31; Osteometrics, Inc.) interfaced with Optronics 3-chip color camera and an Olympus BX60 microscope with epifluorescent light (Leeds Instruments, Irving, TX). All nomenclature for cancellous histomorphometry followed previously established standards (26).

### 2.8 Mechanical testing

Specimens were brought to room temperature and all tests were performed on hydrated specimens using a material testing machine (Instron 3345, Norwood, MA) with a 1000-N load cell. Load and displacement data were collected during tests (at 10 Hz) and analyzed using Bluehill software (Instron Bluehill) and a custom-written Matlab program.

#### 2.8.1 Reduced platen compression (RPC) testing

The metaphyseal region of the tibia was tested for changes in cancellous bone material properties using a reduced platen compression (RPC) test as previously described (27). Briefly, a Well Diamond Wire Saw (Model 3242, Norcross, Georgia) was used to machine a 2-mm-thick cross section from the proximal tibia, which included both cortical and cancellous bone distal to the primary spongiosa of the metaphysis. High-resolution photographs were taken from both sides of each section to determine the appropriate platen size to use for each specimen, such that the platen contacted only cancellous bone and not the cortical shell. Once the specimen was positioned properly between two platens, quasi-static loading was applied with a 100 N load cell at 0.25 mm/min and data were recorded at 20 Hz. Cancellous bone material properties were estimated assuming uniaxial compression as if the cancellous bone material constituted a completely isolated cylindrical specimen, using equations described previously (27).

#### 2.8.2 Three-point bending to failure

Mechanical properties of the femoral midshaft were evaluated using load-to-failure three-point bending tests. The anteroposterior (AP) and mediolateral (ML) surface diameters of each femur were measured at midshaft and the bone was then placed posterior side down on custom-built metal pin (D =3 mm) supports (L =15 mm). The loading pin was centered above the lower supports and contacted the anterior surface at the midpoint of the specimen (mid-diaphysis). Quasi-static loading was applied at the mid-diaphysis to the medial surface under displacement control (2.54 mm/min) with a 1000 N load cell until complete fracture, with a sampling rate of 50 Hz. Load-displacement curves were analyzed to determine the structural variables of ultimate load (highest resistive force achieved, in N) and stiffness (in N/mm), the latter of which defined as the slope of the elastic linear portion of the loading curve. Yield force (in N) was defined as the point where the load-displacement curve transitions from a linear to non-linear curve. Functionally, this was identified as the intersect between the linear portion of the load displacement curve (defining stiffness) and a line with 97% the slope of the stiffness. Material properties were estimated by normalizing structural properties for the bone geometry using the bending cross-sectional moment of inertia (estimated as half the polar CSMI from pQCT), bone diameter (measured by calipers), and the support span distance (L=15 mm). Equations derived from standard beam theory were applied to estimate the material properties of elastic modulus (EM) and ultimate stress (US) as described previously (28).

#### 2.8.3 Femoral neck compression test

Mechanical properties of the femoral neck were assessed via an axial loading testing machine (Instron 3345, Norwood, MA) with a 1000 N load cell. Right proximal femora were settled vertically into a fitted hole in a custom-made aluminum plate fixture (½-inch thick) prior to testing. Using a 10-mm cylindrical platen, a quasi-static load was applied to the femoral head parallel to the axis of the shaft of the femur. A displacement of the platen was maintained at 2.54 mm/min until fracture occurred. Raw data was collected at 20 Hz as load versus displacement curves and analyzed using Bluehill software a custom-written Matlab program. Maximal force (N) was recorded and stiffness (N/mm) was calculated in a similar manner as applied to the femoral midshaft.

### 2.9 Statistical analyses

The data are reported as mean ± standard deviation (SD) for each group; statistical analyses were performed using PRISM (V9.0). Statistical differences between the experimental groups for end-point data (uterine weight; serum estradiol, IGF-1 and leptin; and histomorphometric outcomes) were evaluated using a two-way ANOVA, with Tukey *post-hoc* testing applied when there was a significant interaction term. A one-way ANOVA was utilized to assess differences between treatment groups and baseline control histomorphometry values. Longitudinal data (energy available, body mass, DEXA data) were analyzed by two-way ANOVA for 12-week values, with Bonferroni-corrected t-tests to verify differences from 0-week values. Correlation analyses were performed to assess the relationship (r^2^) between IGF-1 and total body BMC as well as serum leptin and fat mass. P values less than 0.05 were considered statistically significant. P values for main and interaction effects from 2-way ANOVA are reported; all inter-group comparisons as determined by Tukey *post-hoc* tests after ANOVA are significant at p < 0.05, and hence p values are not repeated in the results text.

## 3 Results

### 3.1 Equivalent reductions in energy availability (EA) were achieved in sedentary and exercising energy-restricted groups; exercise modified changes in body mass during moderate energy restriction

**Figure 1:**
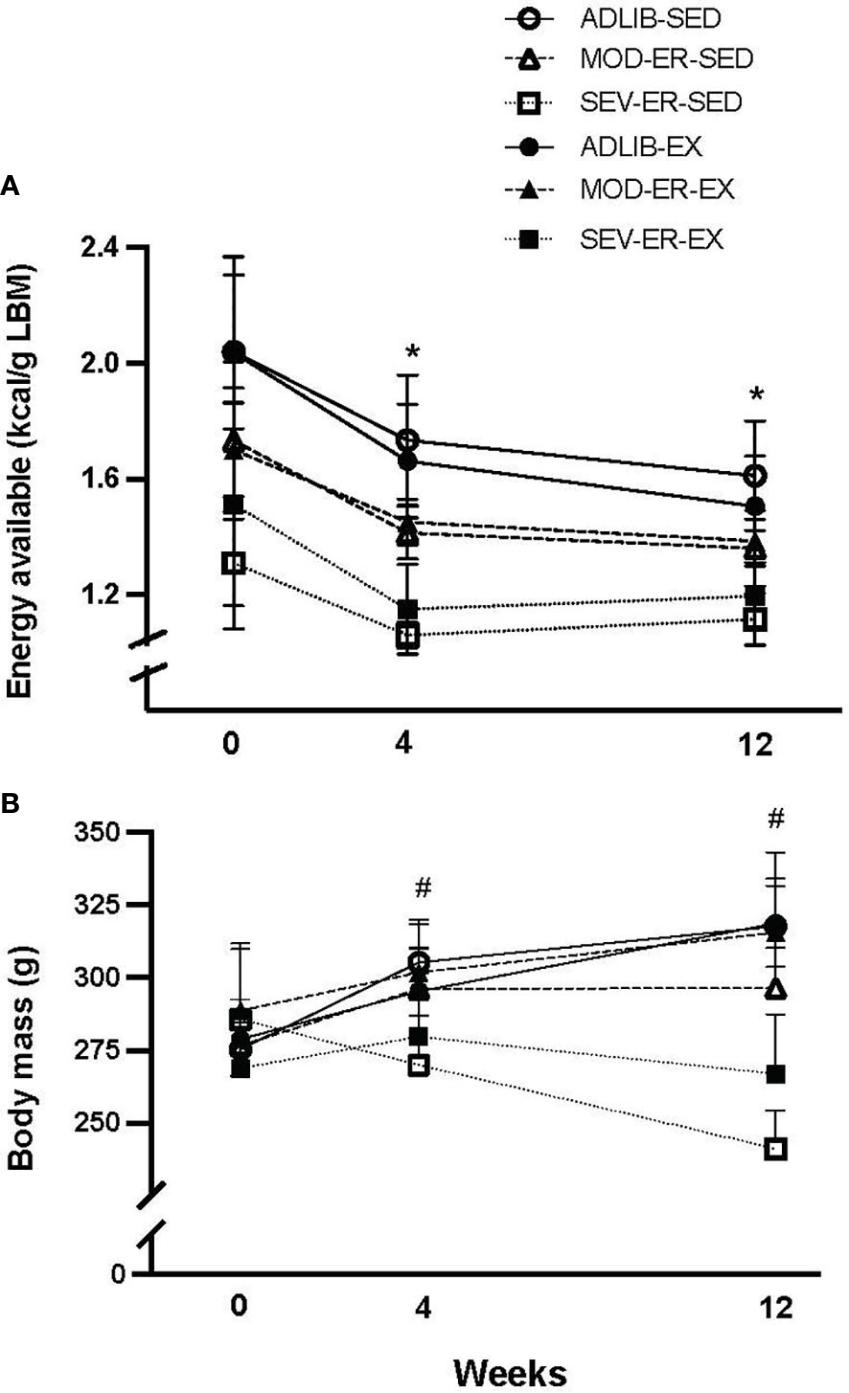
Equivalent reductions in energy availability (kcal/g lean body mass **(A)** were achieved in sedentary (SED) and treadmill trained (EX) groups subjected to moderate (MOD) and severe (SEV) energy restriction (ER) over the course of the experiment. Each data point reflects a group average of the preceding week’s data based on food intake and computed exercise expenditure for each individual rat. Two-way ANOVA’s within time point confirm that energy availability for diet-matched SED and EX groups are not different from each other. *p < 0.05 vs 0-week value within group by Bonferroni-corrected t-tests. Body mass **(B)** changed over time differently across groups over the 12-week experiment; at 12 weeks there was a significant main effect of exercise, evidenced in higher body mass in MOD-ER and SEV-ER exercising animals than noted in sedentary ER groups. # p < 0.03 vs. 0-week value for ADLIB and MOD-ER, but not SEV-ER, groups; the latter exhibited non-significant changes in body mass over the 12 weeks.

During the experimental period several animals were euthanized due to tumor growths from the following groups: one from ADLIB-EX, one from ADLIB-SED, two from MOD-ER-EX, and two from MOD-ER-SED. Both ADLIB*-*fed groups consumed similar levels of calories during the 12-week protocol (**Figure 1A**) and had similar levels of EA/g lean mass. Over the first four weeks, ADLIB-fed animals had declines in food intake resulting in 15-18% declines in EA; the corresponding declines in EA for MOD-ER and SEV-ER groups were 17% and 21.5%, respectively. EA for each group then remained stable for the remainder of the experiment. There was a main effect of energy restriction (p< 0.001) at each of the 3 time points, as intended by the study design; there was no main effect of exercise nor a significant interaction. When calculated over the entire 12 weeks, the MOD-ER groups had ~14% lower EA (-16.3% and -12.4% for SED and EX rats, respectively) and the SEV-ER groups had ~30% lower EA (-35.1% and -25.7% for SED and EX rats, respectively) compared to their respective ADLIB controls. There were no significant differences in EA (kcal/g lean body mass) between SED and EX groups within each level of energy restriction within each time point (**Figure 1A**), (i.e., no main effect of EX) as determined by 2-way ANOVA.

ADLIB sedentary and exercised groups exhibited consistent increases in body mass (**Figure 1B** over the duration of the 12-week protocol (+16% and +15%, respectively, by 12 weeks), verifying that the ADLIB-EX rats increased their food intake to compensate for the increase in caloric expenditure (**Figure 1B**). MOD-ER-EX animals exhibited similar body mass values to those of ADLIB groups, whereas MOD-ER-SED rats exhibited no further body mass gains after 4 weeks. By 12 weeks, SEV-ER-SED and SEV-ER-EX groups exhibited absolute declines in body mass (-16% and -1%, respectively) versus 0 week values. As expected, there was a main effect of ER on body mass and 4 and 12 weeks (p < 0.0001); at 12 weeks, there was also a main effect of EX (p < 0.008), reflected by the higher body mass values for exercising MOD-ER and SEV-ER animals vs EA-matched sedentary groups.

### 3.2 Treadmill exercise mitigated reductions in both fat and lean mass with graded energy restriction

As expected, there was a main effect of ER (p < 0. 0001) on lean and fat mass (**Table 1**) after 12 weeks; in addition, there was a main effect of EX (p < 0.008) on lean, but not fat, mass. Exercising groups exhibited higher lean mass values than did EA-matched sedentary animals. A significant interaction (ER*EX; p = 0.045) was observed for fat mass, as exercise training significantly altered the type of weight gained over the experiment. Fat mass declined in both SED and EX animals with increasing ER, but exercised rats exhibited a 28% increase in fat mass with MOD-ER (vs. a 10% decline in MOD-ER-SED rats). With SEV-ER, fat mass declined over time in both SED and EX rats, but the latter group had a 4-fold greater fat mass vs. SED rats at 12 weeks. Notably, means for fat mass were significantly lower in both sedentary and exercised SEV-ER groups vs. both MOD-ER and ADLIB-fed matched groups.

**Table 1:** Measures of lean and fat mass and total body bone mass [bone mineral content (BMC)] over 12 weeks of matched restriction of energy availability between sedentary and exercise groups.

		ADLIB		MOD-ER		SEV-ER	
		SED	EX	SED	EX	SED	EX
Fat mass (g)	Wk 0	41.9 ± 15.1	39.8 ± 10.0	43.8 ± 13.0	37.3 ± 11.8	49.4 ± 16.2	34.6 ± 10.4
	Wk 12 ^Ψ,π^	66.8 ± 14.7 ^a*^	53.5 ± 18.4^ab^	36.0 ± 12.2 ^bc^	47.0 ± 15.6 ^abc^	4.2 ± 5.5 ^e*^	15.6 ± 17.5 ^de*^
	% change	+74.2	+34.7	-10.4	+27.6	-89.3	-58.3
Lean mass (g)	Wk 0	231.0 ± 19.6	227.9 ± 10.6	226.9 ± 13.7	233.0 ± 16.1	217.2 ± 6.7	223.6 ± 19.0
	Wk 12 ^Ψ,ϕ^	237.4 ± 14.9	250.8 ± 14.4 ^*^	245.8 ± 15.1 ^*^	251.4 ± 21.8 ^*^	208.0 ± 7.2 ^*^	239.2 ± 11.9 ^*^
	% change	+3.0	+10.1	+8.5	+7.8	-4.1	+7.4
Total BMC (g)	Wk 0	9.8 ± 0.7	9.7 ± 0.7	9.7 ± 0.6	10.0 ± 0.9	9.4 ± 0.6	9.6 ± 0.4
	Wk 12 ^Ψ,ϕ^	11.1 ± 0.5 ^*^	11.1 ± 0.9 ^*^	10.5 ± 0.6 ^*^	10.9 ± 0.7 ^*^	8.9 ± 0.5 ^*^	10.0 ± 0.6 ^*^
	% change	+13.7	+15.2	+7.5	+8.8	-5.8	+4.5

Values are means ± standard deviations; group n’s = 7-10. Two-way ANOVA’s within each time point were performed; where there is a significant interaction, groups sharing the same letter are not significantly different by Tukey post-hoc tests. No significant differences were detected among groups for all variables at baseline (Wk 0). Ψ = main effect of energy restriction, p ≤ 0.0001; ϕ = main effect of exercise, p < 0.008; π = interaction ER*EX, p = 0.045. * p < 0.05 vs. Wk 0 value. Percent change values are means of individual rat % change over the 12 weeks.

### 3.3 Reduced EA had a significant impact on total bone mass; this effect was modified by exercise

There were significant main effects for both ER (p< 0.0001) and EX (p =0.008) on total body BMC (**Table 1**), but no interaction effect. Both ADLIB groups experienced significant gains (+13-15%) in total body BMC after twelve weeks (**Table 1**). Graded energy restriction produced step-wise reductions in the gain of total BMC or, in the case of SEV-ER-SED rats, an absolute loss (-7%) over 12 weeks. Severe energy restriction resulted in a significantly lower total bone mass after twelve weeks (-18%) in SED rats vs ADLIB-SED controls. Exercised animals on average had higher total bone mass than EA-matched sedentary groups.

### 3.4 Energy restriction resulted in reductions of bone mass at the proximal tibia and femoral neck; exercise training had a positive impact on femoral neck BMC even in energy-restricted groups

**Figure 2:**
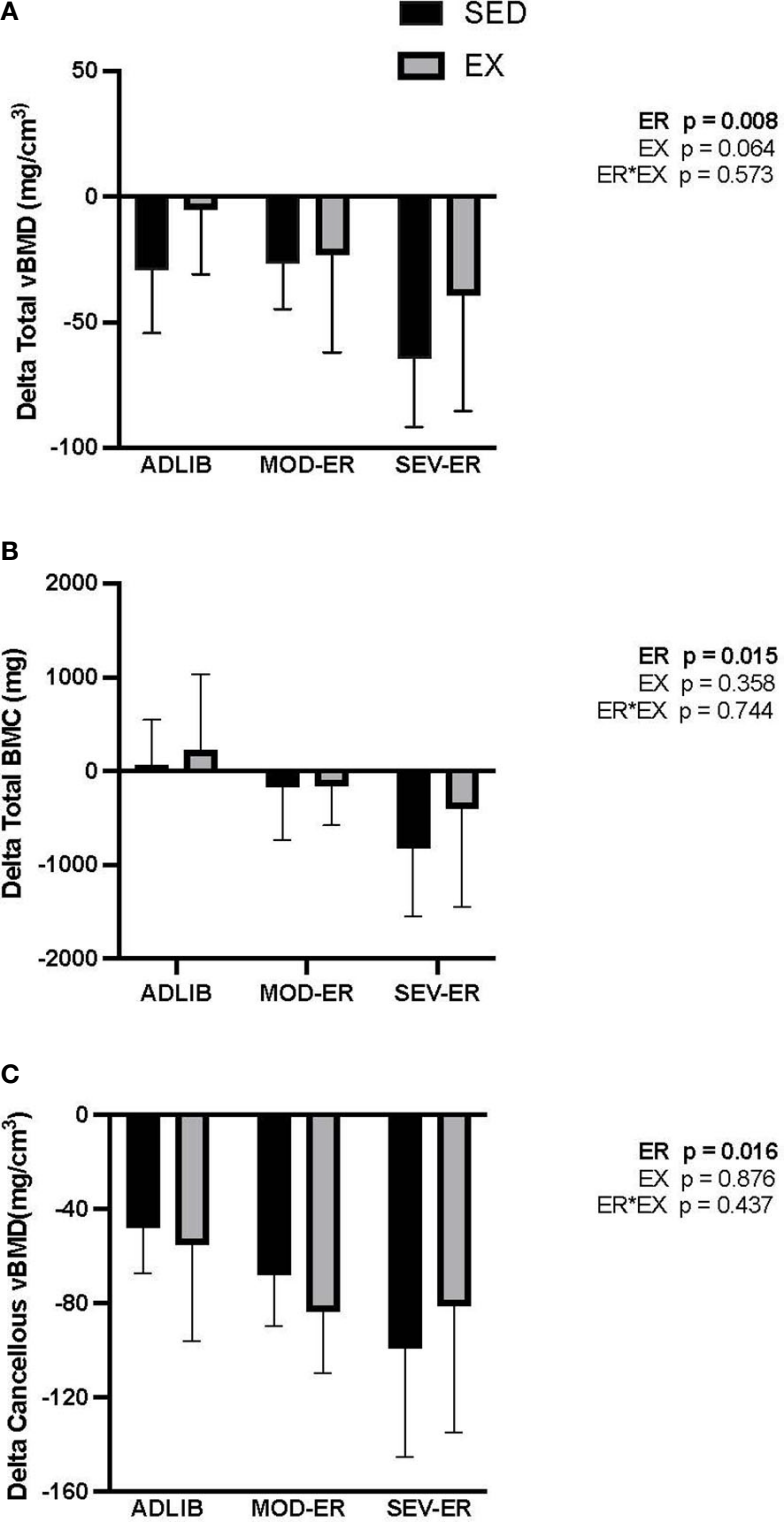
Graded energy restriction (ER) results in progressively greater decrements in volumetric BMD **(A)** and BMC **(B)**, but not for cancellous vBMD **(C)**, at the proximal tibia after 12 weeks, with no evidence for an impact of treadmill exercise (EX).

**Figure 3:**
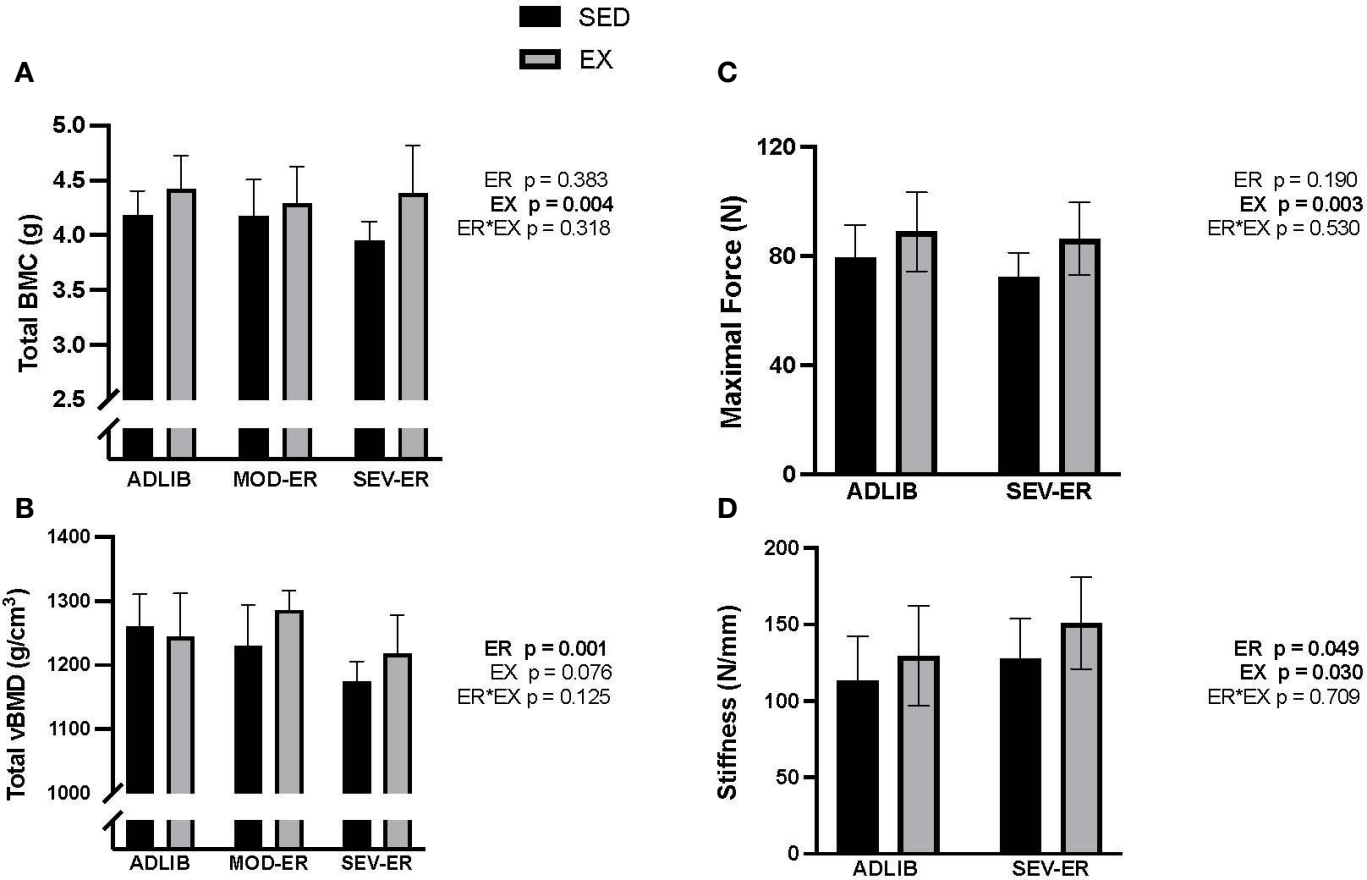
Exercise (EX) exerted positive impacts on femoral neck total BMC **(A)**, while energy restriction (ER) induced significant declines in femoral neck total vBMD **(B)** in sedentary (SED) rats, while maximal force **(C)** and stiffness **(D)** as determined during axial compression, exhibited small gains with exercise and/or severe ER.

ADLIB-fed animals exhibited an age-related loss over the 12 weeks in total (cortical shell plus cancellous compartment) and cancellous vBMD at the proximal tibia (**Figures 2A, C**), as assessed *in vivo* by pQCT scans. Significant main effects for ER were observed for delta total vBMD (p = 0.008), delta total BMC (p = 0.015) (**Figure 2B**) and delta cancellous vBMD (p = 0.016) at the proximal tibia. [Delta values were computed as the difference between 12-week and 0-week (baseline) values for each variable; negative values thus represent a loss over the 12 weeks]. Moderate and severe ER produced stepwise increases in delta vBMD and BMC, with the largest impact observed in SEV-ER groups. There was no main effect of exercise on the 3 outcomes at the proximal tibia, nor any significant interactions. Conversely, at the femoral neck there were main effects for EX (p = 0.004), but not for ER, on total BMC (**Figure 3A**); conversely, there was a significant main effect for ER (p = 0.001), but not for EX, on total vBMD (**Figure 3B**). Severe ER resulted in 5.6% and 6.8% declines (vs. ADLIB controls) in total BMC and vBMD, respectively, at the femoral neck for SED rats. There were no main effects of exercise or energy restriction nor significant interactions for femoral neck cancellous vBMD, cortical thickness, or total cortical area (data not shown).

### 3.5 Prolonged energy restriction did not impair bone mechanical properties; exercise improved femoral neck structural properties

Given the variability inherent in mechanical testing of bone, we limited mechanical testing to samples from ADLIB and SEV-ER groups. Testing of the mid-femur with traditional 3-point bending to failure revealed no significant main effects of ER nor EX on cortical bone structural or material properties (**Table 2**). There was a trend (p = 0.074) for a main effect of exercise on mid-shaft femur stiffness. Similarly, there were no main effects in material properties of distal femur cancellous bone tested with reduced platen compression testing (**Table 2**). Interestingly, a significant main effect for ER (p = 0.049) was detected for femoral neck stiffness and a significant main effect of EX for both FN maximal force (p = 0.003) and stiffness (p = 0.03) and (**Figures 3C, D**). After 12 weeks, maximal force at the femoral neck was 20% higher in SEV-ER-EX rats vs. SEV-ER-SED values; stiffness at this bone site was also 18% higher in exercising vs sedentary rats on SEV-ER.

**Table 2:** Mechanical properties of the mid-shaft femur (3-point bending to failure) and the distal femur metaphysis (reduced platen compression testing) after 12 weeks of matched restriction of energy availability between sedentary and exercise groups.

	ADLIB		SEV-ER	
	SED	EX	SED	EX
Mid-femur cortical				
Ultimate load (N)	177.7 ± 6.0	196.6 ± 10.0	177.1 ± 4.9	182.3 ± 8.2
Stiffness (N/mm)	432.2 ± 28.4	491.8 ± 38.5	444.8 ± 28.1	515.0 ± 46.6
Yield force (N)	140.5 ± 9.8	166.0 ± 7.7	142.5 ± 7.3	143.9 ± 10.3
Ultimate stress (MPa)	151.6 ± 6.1	159.3 ± 6.9	154.3 ± 4.6	151.7 ± 6.9
Elastic modulus (GPa)	4.4 ± 0.4	4.7 ± 0.4	4.6 ± 0.3	5.0 ± 0.6
Distal femur cancellous				
Ultimate stress (MPa)	2.6 ± 1.8	3.1 ± 1.4	2.0 ± 1.1	2.6 ± 1.5
Strain at Ult Stress (x 10^-3^)	2.1 ± 1.1	4.1 ± 3.1	11.2 ± 13.5	3.3 ± 2.7
Elastic modulus (MPa)	59.0 ± 30.2	58.0 ± 26.4	40.7 ± 27.9	51.8 ± 20.6

Values are means ± standard deviations; n =9-11/group for mid-femur 3-point bending data, n=6-8 for distal femur RPC testing. Two-way ANOVA’s were performed; for all variables, no significant main or interaction effects were detected.

### 3.6 Cancellous microarchitecture, bone cell activity measures, and marrow adipocyte density were impacted by energy restriction but minimally impacted by exercise

There was a main effect of ER on cancellous bone volume (%BV/TV), trabecular thickness (Tb.Th) and osteoid surface (% OS/BS) (p = 0.034, 0.002, and 0.026, respectively) (**Table 3**). There was a main effect of EX only on % OS/BS (p = 0.046) and no significant interactions for any histomorphometric outcome. Graded ER produced progressively lower %BV/TV values at the proximal tibia metaphysis, with 12% and 27% lower values observed after 12 weeks in MOD-ER and SEV-ER sedentary rats, respectively, versus ADLIB fed controls (**Table 3**). A concurrently lower (-18%) trabecular thickness (Tb.Th) and a greater (+33%) trabecular spacing (Tb.Sp) was observed in SEV-ER-SED rats vs ADLIB controls. Tb.Th values for SEV-ER-EX rats were not significantly lower than baseline values, whereas the SEV-ER-SED mean was 15% lower vs. baseline rats’ mean.

**Table 3:** Proximal tibia cancellous microarchitecture and indices of bone formation/resorption before and after 12 weeks of matched restriction of energy before availability between sedentary and exercise groups.

	BASELINE CONTROL	ADLIB		MOD-ER		SEV-ER	
		SED	EX	SED	EX	SED	EX
BV/TV (%)^ψ^	29.1 ± 4.9	24.5 ± 3.4	23.1 ± 5.1^*^	21.6 ± 4.9 ^*^	24.2 ± 6.7	18.1 ± 6.2 ^*^	20.0 ± 5.1 ^*^
Tb.Th (µm) ^ψ^	48.1 ± 4.5	49.8 ± 5.7	51.4 ± 4.8	48.5 ± 6.6	52.3 ± 9.0	40.9 ± 4.2^*^	44.8 ± 7.3
Tb.Sp (µm)	120.1 ± 23.0	155.3 ± 20.6	179.0 ± 45.6 ^*^	181.1 ± 34.4 ^*^	177.9 ± 67.8 ^*^	206.6 ± 78.3 ^*^	185.8 ± 38.0 ^*^
Tb.N (1/mm)	6.0 ± 0.9	4.9 ± 0.5 ^*^	4.5 ± 0.8 ^*^	4.4 ± 0.6 ^*^	4.6 ± 1.0 ^*^	4.4 ± 1.2 ^*^	4.4 ± 0.8 ^*^
OS/BS (%)^ψ,^^ϕ^	1.6 ± 1.5	1.8 ± 0.7	2.6 ± 1.8	2.9 ± 1.9	3.7 ± 2.2	1.2 ± 0.5	2.4 ± 1.6
ObS/BS (%)	0.7 ± 0.9	0.6 ± 0.3	1.1 ± 1.1	1.3 ± 1.0	1.4 ± 1.1	0.5 ± 0.2	1.0 ± 0.9
OcS/BS (%)	2.3 ± 1.2	2.2 ± 2.3	1.9 ± 1.2	2.1 ± 2.0	1.9 ± 1.4	1.7 ± 1.4	1.8 ± 1.1

Values are means ± standard deviations; group n’s =6-11. Two-way ANOVA’s were performed. There were no significant interactions. Ψ = main effect of energy restriction, p < 0.03; ϕ = main effect of exercise, p < 0.05. * = p < 0.05 vs. baseline group value by Bonferroni-corrected t-test. BV/TV, % bone volume/tissue volume; Tb.Th, trabecular thickness; Tb.Sp, trabecular spacing; Tb.N, trabecular number; OS/BS, % osteoid surface/total bone surface; ObS/BS, % osteoblast surface/total bone surface; OcS/BS, % osteoclast bone surface/total bone surface.

**Figure 4:**
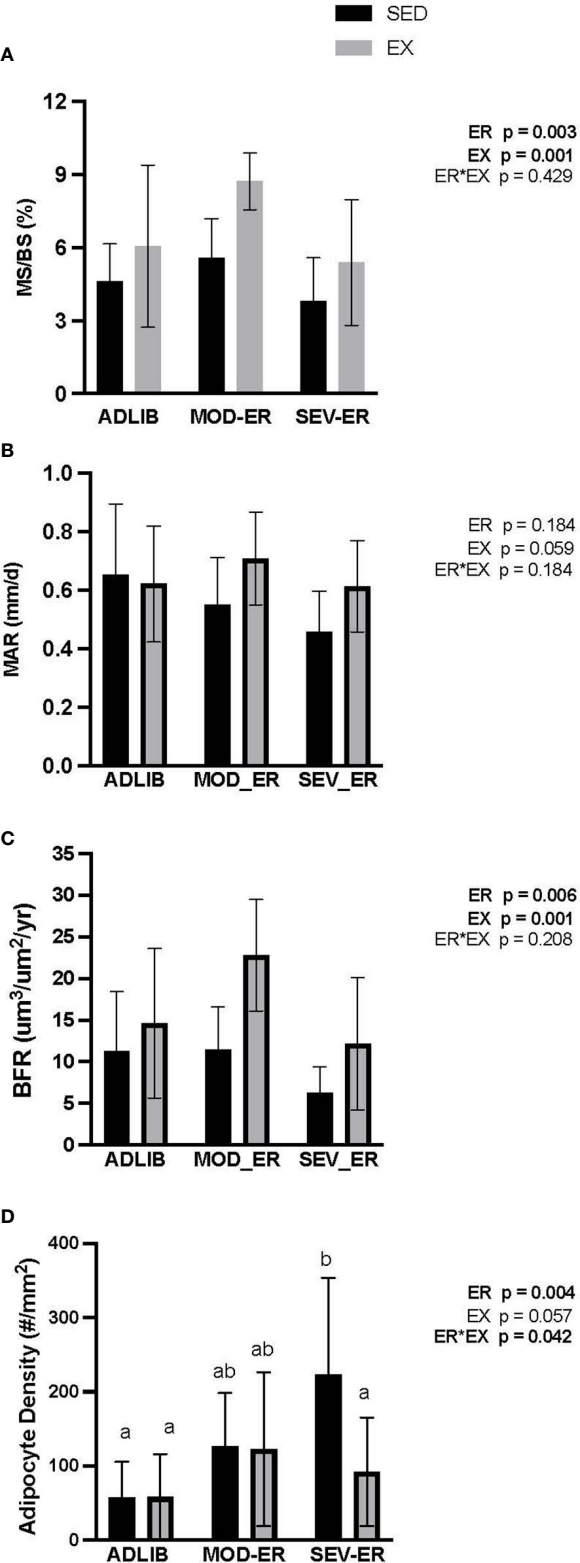
Treadmill exercise (EX) combined with moderate energy restriction (MOD-ER) enhances indices of bone formation in proximal tibia cancellous bone, while marrow adipocyte density increases with severe (SEV) energy restriction in sedentary (SED) rats only. **(A)** Mineralized Surface/Bone Surface (MS/BS, %). **(B)** Mineral Apposition Rate (MAR, %). **(C)** Bone Formation Rate/Bone Surface (BFR/BS, %). **(D)** Adipocyte Density (# adipocytes/mm^2^). Group means not sharing the same letter are significantly different from one another (p<0.05).

There were no main effects of ER or EX on osteoblast nor osteoclast surfaces (% ObS/BS and % Oc/BS, respectively) in proximal tibia cancellous bone (**Table 3**). Dynamic histomorphometric analyses of calcein-labelled proximal tibia sections revealed no main effects of ER or EX on mineral apposition rate (MAR) (**Figure 4B**). However, significant main effects for both ER and EX were detected on the extent of mineralizing surfaces (%MS/BS, reflecting osteoblast recruitment; p = 0.003 and 0.001, respectively) (**Figure 4A**) and on bone formation rate (BFR; p = 0.006 and 0.001) (**Figure 4C**). Interestingly, MOD-ER-EX animals exhibited ~50% larger numerical values for %MS/BS and BFR vs. values in ADLIB-EX rats. All %MS/BS and BFR values were numerically higher for all EX vs SED group. There was a main effect of ER on adipocyte density in the proximal tibial metaphysis (p = 0.004) and a significant ER*EX interaction (p = 0.042). Graded ER resulted in stepwise increases in adipocyte density in the proximal tibia metaphysis for SED but not EX groups (**Figure 4D**). SEV-ER in SED animals produced a > 4-fold higher adipocyte density vs. ADLIB controls; endurance training abolished this response in SEV-ER-EX animals.

### 3.7 Reduced energy availability negatively affects estradiol levels and uterine weight in sedentary but less so in exercised animals

**Figure 5:**
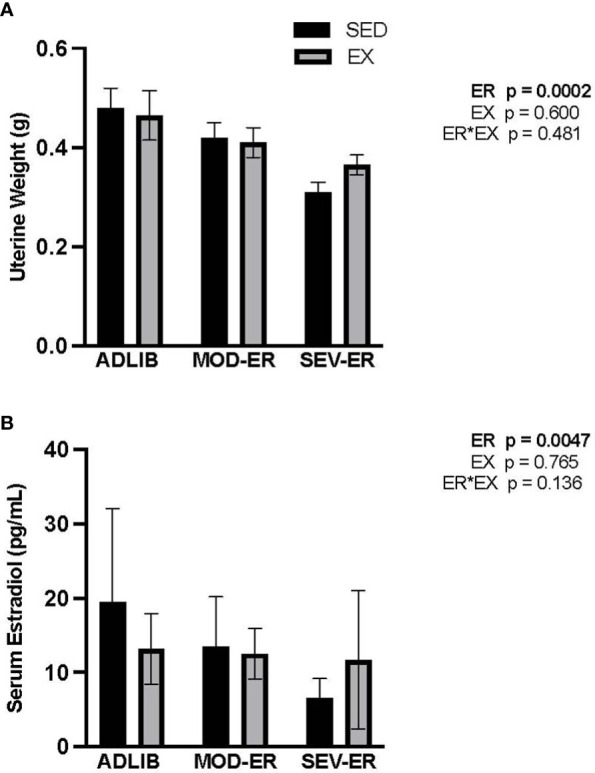
Energy restriction (ER) negatively affects markers of estrogen status, with no apparent impact of exercise. **(A)** Uterine weight (g); **(B)** Serum estradiol (pg/ml).

There were highly significant main effects for ER on both uterine weight (p = 0.0002) and serum estradiol (p = 0.0047), but no ER*EX interactions nor any impact of EX (**Figure 5**). In ADLIB*-*fed animals, there were no differences in uterine weight and serum estradiol between SED and EX groups. Severe ER produced lower estradiol levels (-63%) and uterine weight values (-36%) vs. ADLIB-SED. A qualitative assessment of estrus cycles (from vaginal smear data) reveals an increase in the proportion of animals with either irregular cycles or cessation of estrus with increasing levels of ER, which effect was most pronounced in exercising groups (**Supplementary Figure 1**).

### 3.8 Exercise training status modifies alterations in endocrine markers of energy metabolism induced by graded energy restriction

**Figure 6:**
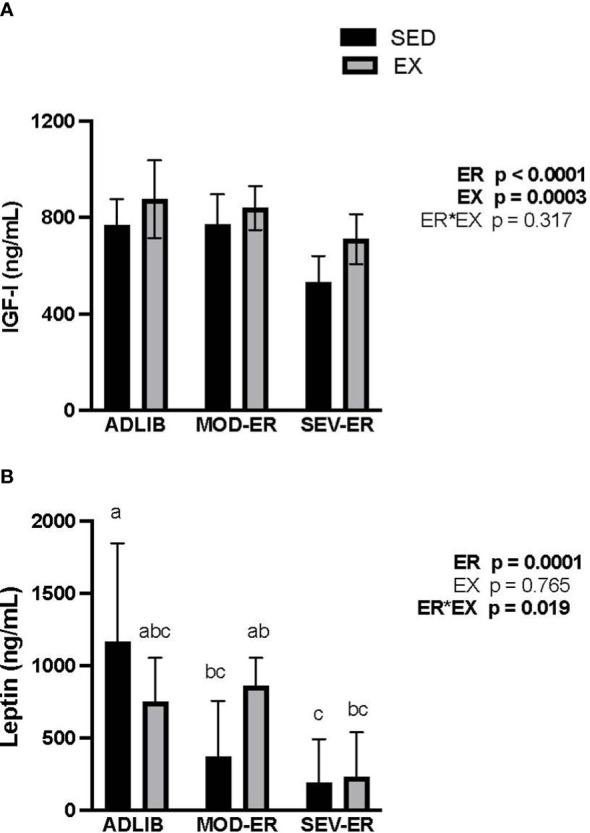
Energy restriction (ER) exerts significant negative impacts on serum insulin-like growth factor (IGF-1, ng/mL; **(A)**) and on serum leptin (ng/mL; **(B)**). The impact of MOD-ER on serum leptin appears to be mitigated by treadmill training (EX) when energy availability is matched between sedentary (SED) and EX groups. Group means not sharing the same letter within time point are significantly different from one another (p<0.05).

At baseline, there were no significant differences in serum IGF-1 and leptin values among the six experimental groups (data not shown). After 12 weeks there were main effects of ER (p < 0.0001) and of EX (p = 0.0003) on IGF-1 (**Figure 6A**). In ADLIB*-*fed animals EX did not result in values higher than those observed in ADLIB-SED animals (**Figure 6A**). Severe ER resulted in 31% lower serum IGF-1 values vs. ADLIB-SED rats. Exercise training resulted in higher serum IGF-1 values in all EA-matched groups; e.g., exercised SEV-ER values at 12 weeks were 25% higher than those in sedentary SEV-ER rats. By contrast, there was no main effect of EX but a significant main effect of ER (p = 0.0001) on serum leptin levels, with a significant ER*EX interaction (**Figure 6B**). Serum leptin levels were 68% and 84% lower in MOD-ER and SEV-ER sedentary rats, respectively, vs. ADLIB sedentary animals. Exercise largely prevented the reductions in serum leptin, which is consistent with the smaller reductions in fat mass observed in exercising SEV-ER animals. There were moderately strong correlations between IGF-1 and total body BMC values at 12 weeks for both pooled sedentary (r^2 = ^0.69, p<0.0001) and pooled exercised (r^2 = ^0.58, p<0.001) animals. Leptin values at 12 weeks correlate significantly with fat mass for both pooled exercised (r^2 = ^0.66, p < 0.01) and pooled sedentary (r^2 = ^0.67, p<0.01) rats, but not with total body BMC.

## 4 Discussion

Our objective was to determine whether the means of achieving graded reductions in energy availability (with or without exercise) alters the bone and endocrine response to this stressor. We hypothesized that combining moderately vigorous endurance exercise training with reduced dietary energy intake would lessen the negative impact of reduced energy availability on bone parameters through both attenuated changes in bone-relevant endocrine factors and increased mechanical loading on bone. Our results confirm the significant negative effects of severe energy restriction on total body bone mass (BMC), cancellous bone volume at mixed bone sites (proximal tibia), and key regulatory endocrine factors (estradiol, IGF-1, and leptin) previously documented by us and other research groups in sedentary rodents. When the same degree of reduced energy availability (~30%) was achieved with a mix of exercise training and reduced energy intake, the negative impact on total bone mass relative to ADLIB controls appeared to be mitigated. Exercising animals on severe ER gained 4.5%, while sedentary SEV-ER rats lost 5.8%, total body BMC after 12 weeks.

In humans, prolonged reduced energy availability generally has large negative impacts on cancellous bone mass and microarchitecture at mixed bone sites (lumbar spine, long bone metaphyses) (25). In this animal model, the impact of exercise on metaphyseal bone sites during graded reductions in energy availability was less pronounced than that on total skeletal mass. Exercise training had no significant impact on the loss of proximal tibia bone mass seen with both levels of energy restriction. We note, however, that for the exercising SEV-ER group the magnitudes of loss (delta values) in total vBMD and total BMC were 39% and 51% smaller, respectively, than in matched sedentary groups (**Figure 2**). All exercised groups exhibited higher bone mineral content at the femoral neck, another mixed bone site.

The partial protection against bone loss provided by our endurance running paradigm during more severe reduced energy availability might be explained by the higher serum IGF-1 values observed in SEV-ER-EX rats vs SEV-ER-SED animals. We found positive correlations between serum IGF-1 (r^2^ of 0.58-0.69) and total body BMC in both sedentary and exercising groups. Synthesis of IGF-1 and its availability in the systemic circulation is tightly related to energy intake. Therefore, large reductions in energy available to an organism predictably lead to significant decrements in IGF-1 levels (29, 30) and often a concurrent decrease in osteoblast activity (31). Multiple investigations in humans confirm strong relationships between reduced IGF-1 during reduced EA and the bone deficits observed (12, 13). While reduced EA did result in significantly lower IGF-1 values versus ADLIB controls, serum IGF-1 for exercising rats was elevated in all EA matched groups versus sedentary animals. Serum IGF-1 values were lower For sedentary SEV-ER rats vs those in ADLIB rats (-31%) but also lower than their week 0 values (-19%) (data not shown). We cannot determine with our study design if this better preservation of circulating IGF-1 in SEV-ER-EX rats is due to the treadmill exercise regimen itself and/or to less severe restriction of energy intake.

Hypoestrogenemia and disrupted menstrual/estrus cycling are frequently observed with reduced energy availability in humans and rodents. After 10 weeks of 40% energy restriction in 6-mo-old sedentary female rats, serum estradiol declines by >50% (32). In our experiment, SEV-ER resulted in significantly lower uterine weights vs ADLIB values in both sedentary and exercising female rats (-36 and -22%, respectively). Serum estradiol was significantly reduced (-63%) vs ADLIB controls only in sedentary rats subjected to severe ER. Our qualitative results on estrus cycling (**Supplementary Figure 1**) indicate increasingly disrupted estrus cycles with graded reductions in energy availability, particularly in exercising animals, which is consistent with findings of previous investigations (33–35). A cross-sectional study of women who were estrogen- and/or energy-deficient and exhibited a wide range of menstrual status disturbance illustrated that tibial bone vBMD, geometry and estimated strength was dependent on energy status, whereas the same outcomes in the radius were dependent on estrogen status, suggesting that the mechanical loading provided by weight-bearing activity can play a significant role in protecting bone against the impact of chronic hypoestrogenemia (12). The classic experiment of Ihle and Loucks (13) imposed graded reductions in energy availability on young women and documented stepwise reductions in bone formation markers and IGF-1 and, at the severest level of reduced energy availability, increases in serum markers of bone resorption, reduced estradiol and disrupted LH pulsatility (15, 36). This tightly controlled experiment lasted only five days, too short a duration to produce detectable changes in bone microarchitecture, geometry and bone mass, all of which impact on the ultimate variable of interest: bone’s resistance to fracture (its mechanical integrity).

A major contribution of this investigation is our finding that moderate and even severe energy restriction, regardless of exercise status, had minimal to no impact on bone mechanical properties assessed at three different bone sites (mid-shaft femur, distal femur and femoral neck), even as bone mass declined at the latter two sites. These findings differ from previous results demonstrating declines in maximal force and stiffness at the mid-femur with 10 weeks of 40% ER in young male mice (17). These disparate findings may reflect a species difference, since others have found declines in long bone mechanical strength and stiffness in older (11-mo-old) but not younger (5-mo-old) rats subjected to 40% energy restriction for 9 weeks (14). Notably, in our study exercise training did exert a positive impact on femoral neck maximal force and stiffness in the context of both energy-replete and energy-deficient states. Exercise appears effective in improving mechanical integrity at this clinically important mixed bone site even in the face of severe ER over 12 weeks.

Strain at ultimate stress in distal femur cancellous bone, an indicator of bone deformation at the applied load corresponding to maximum stress, was >3-fold higher in sedentary vs exercised rats on SEV-ER. This finding is consistent with the larger declines in metaphyseal bone vBMD and BMC observed in sedentary (vs. exercising) rats in this investigation at the neighboring proximal tibia. The high variability in strain at ultimate stress for SEV-ER-SED animals no doubt contributed to the lack of statistical significance for this comparison. Although we observed minimal impact on bone mechanical integrity after 12 weeks of reduced EA, it is clear that the multiple impacts of the more severe level of reduced EA (~30%) induces enough change in bone mass, geometry and cancellous microarchitecture that more prolonged exposures to reduced EA might well impair fracture resistance of bone. In humans, this translates to a higher rate of stress fractures observed in athletes experiencing prolonged reductions in EA (7, 8).

Our data also provide evidence for positive impacts of exercise training even during severe ER on bone formation activity in cancellous bone, with higher values for trabecular thickness, % mineralizing surfaces and bone formation rate in exercising rats vs EA-matched sedentary controls. These results are consistent with higher values for IGF-1 in the exercising animals, given its known anabolic impacts on osteoblast differentiation and activity (31, 37). Interestingly, moderate energy restriction (resulting in ~ 14% reduced energy availability) generally resulted in higher % mineralizing surfaces (MS/BS) and bone formation rate in proximal tibial cancellous bone. Since mineralizing apposition rate (MAR) was unaffected, this finding suggests that moderate ER had more of an impact on maintaining osteoblast differentiation and/or numbers, rather than on osteoblast activity. This speculation is consistent with the large numerical increases in osteoblast surfaces (%ObS/BS) and significant increases in observed in unmineralized osteoid surfaces (%OS/BS) in MOD-ER groups (vs. ADLIB-fed controls). We also note the strong suppression of the 4-fold rise in marrow adipocyte density in exercising animals on SEV-ER, which would support increased differentiation in the direction of osteoblastic precursor cells. These positive changes in rats exposed to moderate reductions in EA, as well as the lack of negative impacts on bone mechanical properties, could be due to reduced systemic inflammation commonly observed with milder caloric restriction. For example, long-term caloric restriction (-25%) without malnutrition in non-obese adults results in significant and persistent inhibition of multiple inflammatory markers like TNF-a and C-reactive protein (38).

Exercise training in our experiment significantly altered the impact of reduced EA on body composition. Moderate energy restriction resulted in a slightly smaller increase in body mass than observed in ADLIB-SED controls; this gain was achieved by an 8% increase in lean mass counterbalancing the 10% decrease in fat mass. Interestingly, exercise training during moderate ER resulted in a small gain (+28%) in fat mass and total body mass. Even with prolonged severe ER, endurance-trained rats maintained total body mass, gained a small amount of lean mass and retained more fat mass than did sedentary SEV-ER animals. Our previous work utilizing comprehensive multiple linear regression analyses on a larger cohort of animals found that both total lean and total fat mass predicted total bone mass (BMC) in skeletally mature female rats subjected to prolonged reduced energy availability (22). These results support the value of including exercise in weight loss efforts in better maintaining lean mass and bone integrity. Interestingly, exercising rats exposed to severe ER in this investigation exhibited less loss of fat mass (-58%) over 12 weeks as compared to EA-matched sedentary animals (-89%). It is possible that the 33% higher serum IGF-I concentrations in the severe ER group may play a role in explaining this finding. Others have reported that mice with a conditional knock-out of both insulin and IGF-I receptors specific to fat tissue exhibit a marked reduction in both white and brown fat mass, suggesting an important role for IGF-I in supporting maintenance of adipose tissue stores (39).

Several published studies have documented negative impacts of exercise during periods of restricted energy intake. In female rats with constant access to a running wheel starting at 4 months of age, 30% food restriction results in significant weight loss, decreased serum leptin and estradiol, and decreased BMC of the femur and tibia (33) after 12 weeks. A similar protocol exposing female mice to 30-50% food restriction starting at 2 months of age documented no gains in total bone mass (BMC) after 8 weeks for both sedentary and running wheel-exposed mice, vs ~20% gains in adlib-fed controls (34). It must be noted that these two protocols involved restricting *all* nutrients (by reducing only volume of chow provided each rat), including bone-critical nutrients like protein and calcium. A more recent protocol utilizing 30% restriction of energy only (using a nutrient-enriched diet similar to our protocol) in female mice starting at 3 months of age with access to voluntary wheel running demonstrated greater bone decrements, particularly in cortical bone (e.g, increased cortical porosity), as compared to sedentary controls fed the same energy-restricted diet after only 6 weeks (40). The lower energy availability in these exercised mice (due to increased energy expenditure with ~ 10 km/d running) vs. that in sedentary mice provided the same restricted diets, as well as in the Mequinion et al. study (34), confounds interpretation of these results vis-à-vis the current study’s findings.

There were a few limitations to our investigation. There were slight variations in macronutrient content among the four custom-made variations on AIN93-M diet; in particular, the purified diets fed to the SEV-ER animals contained slightly more protein and fat (as % of total kcal content) balancing out the reduced carbohydrate content. These variations are fairly small compared to the much larger reductions in energy achieved with our feeding protocols and are unlikely to impact on our results. Secondly, due to the high volume of measurements requiring anesthesia during the last week of the study, exercising groups were not able to complete all days of scheduled exercise treatment, resulting in greater energy availability than intended over that last week. This increased energy availability as compared to SEV-ER sedentary rats may have influenced serum endocrine values measured at 12 weeks to a small degree or even the diminished loss of body fat mass.

In conclusion, our findings suggest that achieving a ~30% reduction in energy availability with combined exercise and energy restriction mitigates the deleterious effects on total body bone mass, lean mass, femoral neck vBMD, and serum IGF-1 seen with energy restriction alone. In addition, a moderate reduction in energy availability (~ -14%) produced surprisingly few deleterious effects on bone integrity, nor did either level of reduced energy availability significantly impact on bone mechanical properties after 12 weeks. These findings are particularly important for long term bone health in highly active populations such as military personnel and athletes who may frequently experience periods of reduced energy availability, intentionally or not.

## Data availability statement

The raw data supporting the conclusions of this article will be made available by the authors, without undue reservation.

## Ethics statement

The animal study was reviewed and approved by Institutional Animal Care and Use Committee, Texas A&M University-College Station.

## Author contributions

SB, MD, and HH designed the original protocol. MD supervised all endocrine assays. SS and KB ran the animal protocol, performed data analyses, and edited the manuscript. CM performed data analyses and edited the manuscript. SL and YS-F performed the bone mechanical testing and performed data analyses. HH supervised all bone mechanical testing. SB, SS, CM, KB, MD, SL, YS-F, and HH approved the final version. All authors contributed to the article and approved the submitted version.

## Supplementary material

Supplementary Figure 1 Characterization of estrus cycles as regular, irregular or acyclic from daily vaginal smears collected over 2 weeks’ time just prior to 0, 4 and 12 weeks of moderate or severe energy restriction in sedentary animals. “Acyclic” designates vaginal smear results indicating no regular repetitions of diestrus, metestrus and estrus phases; “irregular” designates those patterns including 2 or 3 of these phases but without regular timing of phases.

Supplementary Figure 2 Characterization of estrus cycles as regular, irregular or acyclic from daily vaginal smears collected over 2 weeks’ time just prior to 0, 4 and 12 weeks of moderate or severe energy restriction in exercising animals. “Acyclic” designates vaginal smear results indicating no regular repetitions of diestrus, metestrus and estrus phases; “irregular” designates those patterns including 2 or 3 of these phases but without regular timing of phases.
