# Supplementary figures and images for: Exercise training modifies the bone and endocrine response to graded reductions in energy availability in skeletally mature female rodents
Source: Front Endocrinol (Lausanne). 2023 Jun 27;14:1141906. doi: 10.3389/fendo.2023.1141906 (PMC10338226; doi:10.3389/fendo.2023.1141906)

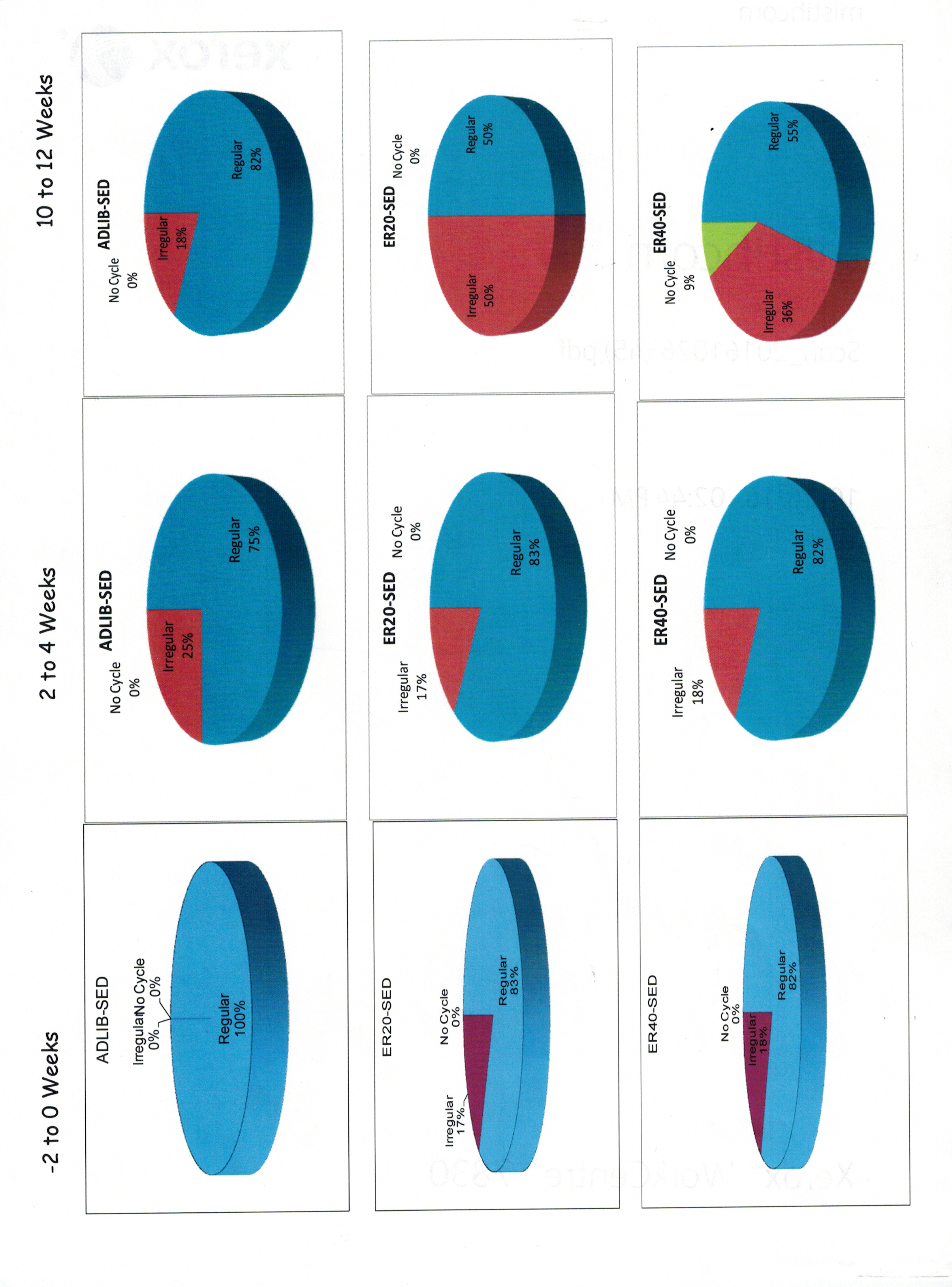

Supplement: Supplementary Figure 1 — Characterization of estrus cycles as regular, irregular or acyclic from daily vaginal smears collected over 2 weeks’ time just prior to 0, 4 and 12 weeks of moderate or severe energy restriction in sedentary animals. “Acyclic” designates vaginal smear results indicating no regular repetitions of diestrus, metestrus and estrus phases; “irregular” designates those patterns including 2 or 3 of these phases but without regular timing of phases. [file Image_1.jpeg]

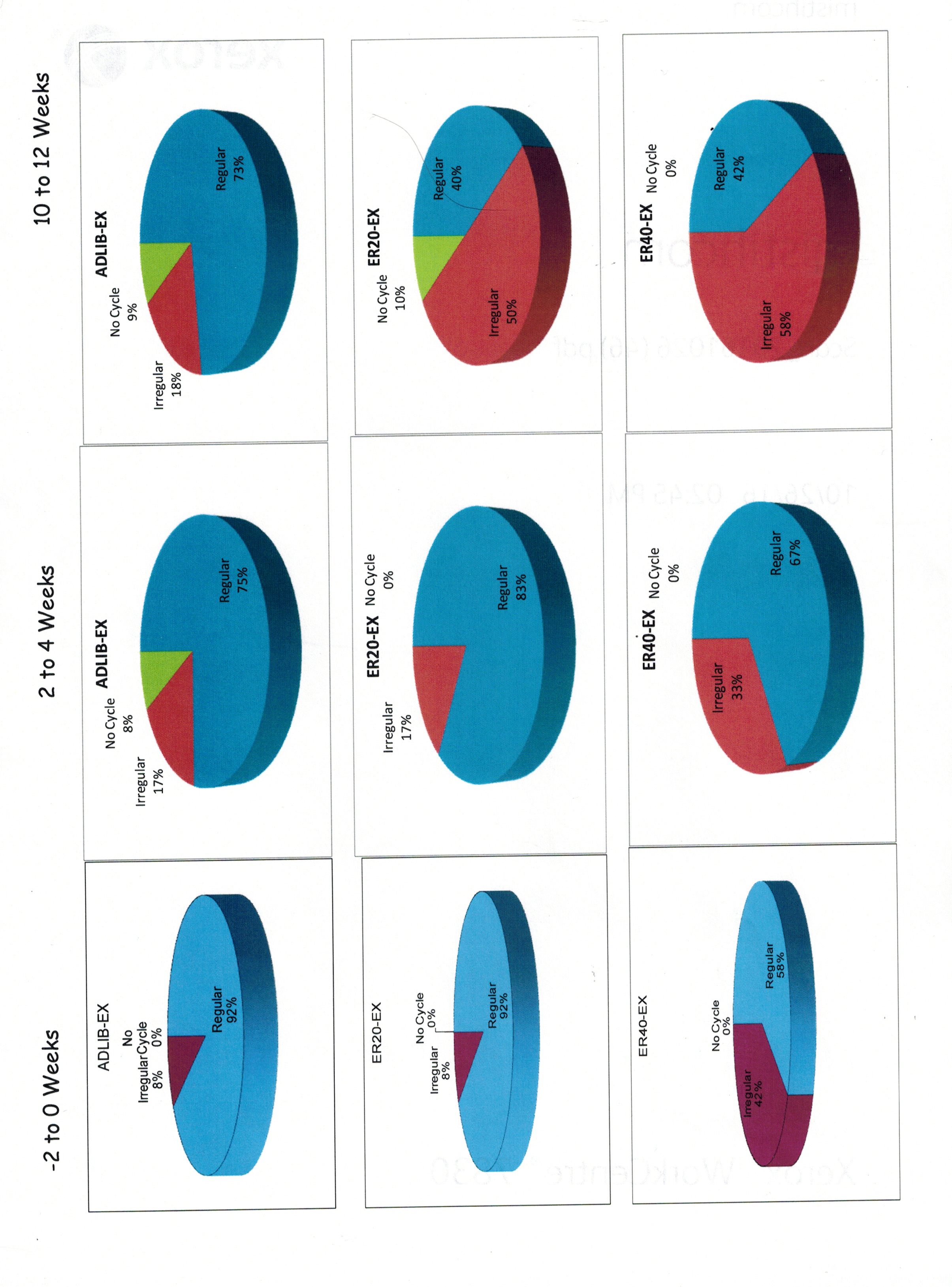

Supplement: Supplementary Figure 2 — Characterization of estrus cycles as regular, irregular or acyclic from daily vaginal smears collected over 2 weeks’ time just prior to 0, 4 and 12 weeks of moderate or severe energy restriction in exercising animals. “Acyclic” designates vaginal smear results indicating no regular repetitions of diestrus, metestrus and estrus phases; “irregular” designates those patterns including 2 or 3 of these phases but without regular timing of phases. [file Image_2.jpeg]
