# Supplementary material for: Exercise training modifies the bone and endocrine response to graded reductions in energy availability in skeletally mature female rodents
Source: Front Endocrinol (Lausanne). 2023 Jun 27;14:1141906. doi: 10.3389/fendo.2023.1141906 (PMC10338226; doi:10.3389/fendo.2023.1141906)
Supplement: Supplementary file 3 [file Table_1.docx]

**Supplementary Table 1**. Macronutrient composition of experimental diets, based on AIN-93M diet (Research Diets, Inc.;

New Brunswick, NJ, USA), expressed as % of total kcal, and energy density

|  | **AIN-93M**  **(ADLIB-SED, ADLIB-EX)** | **-10% KCAL**  **(MOD-ER-EX)** | **-20% KCAL**  **(MOD-ER-SED)** | **-30% KCAL**  **(SEV-ER-EX)** | **-40% KCAL**  **(SEV-ER-SED)** |
| --- | --- | --- | --- | --- | --- |
|  |  |  |  |  |  |
| Protein | 15 | 16 | 18 | 21 | 24 |
| Carbohydrate | 76 | 73 | 70 | 66 | 60 |
| Fat | 9 | 10 | 12 | 13 | 16 |
| Energy (kcal/gm) | 3.85 | 3.83 | 3.81 | 3.79 | 3.76 |
